# Complete Metabolic Response in FDG-PET-CT Scan before Discontinuation of Immune Checkpoint Inhibitors Correlates with Long Progression-Free Survival

**DOI:** 10.3390/cancers13112616

**Published:** 2021-05-26

**Authors:** Timo E. Schank, Andrea Forschner, Michael Max Sachse, Antonia Dimitrakopoulou-Strauss, Christos Sachpekidis, Albrecht Stenzinger, Anna-Lena Volckmar, Alexander Enk, Jessica C. Hassel

**Affiliations:** 1 Department of Dermatology and National Center for Tumor Diseases, University Hospital Heidelberg, 69120 Heidelberg, Germany; alexander.enk@med.uni-heidelberg.de (A.E.); jessica.hassel@med.uni-heidelberg.de (J.C.H.); 2 Department of Dermatology, University Hospital Tübingen, 72076 Tübingen, Germany; andrea.forschner@med.uni-tuebingen.de; 3 Department of Dermatology, Allergology, and Phlebology, Hospital Bremerhaven Reinkenheide, 27574 Bremerhaven, Germany; michael.sachse@klinikum-bremerhaven.de; 4 Clinical Cooperation Unit Nuclear Medicine, German Cancer Research Center, 69120 Heidelberg, Germany; a.dimitrakopoulou-strauss@dkfz.de (A.D.-S.); c.sachpekidis@dkfz.de (C.S.); 5 Department of Pathology, University Hospital Heidelberg, 69120 Heidelberg, Germany; albrecht.stenzinger@med.uni-heidelberg.de (A.S.); anna-Lena.volckmar@med.uni-heidelberg.de (A.-L.V.)

**Keywords:** FDG-PET-CT scan, melanoma, outcome, checkpoint inhibitors

## Abstract

**Simple Summary:**

Immunotherapy is the standard of care in patients harboring metastasized melanoma. However, once further tumor growth is stopped it remains unclear when immunotherapy can be safely ceased. This clinical question is increasingly raised especially in patients with a strong desire to discontinue therapy or in patients who are forced to pause treatment due to severe immune-related side effects. With our study we aim to provide data which may be helpful for clinicians and patients when treatment discontinuation is considered. Further prospective, multicenter studies are needed to further address this important clinical issue.

**Abstract:**

Checkpoint inhibitors have revolutionized the treatment of patients with metastasized melanoma. However, it remains unclear when to stop treatment. We retrospectively analyzed 45 patients (median age 64 years; 58% male) with metastasized melanoma from 3 cancer centers that received checkpoint inhibitors and discontinued therapy due to either immune-related adverse events or patient decision after an (^18^F)2-fluoro-2-deoxy-D-glucose positron emission tomography (FDG-PET) combined with a low-dose CT scan (FDG-PET-CT) scan without signs for disease progression. After a median of 21 (range 1–42) months of immunotherapy an FDG-PET-CT scan was performed to evaluate disease activity. In these, 32 patients (71%) showed a complete metabolic response (CMR) and 13 were classified as non-CMR. After a median follow-up of 34 (range 1–70) months, 3/32 (9%) of CMR patients and 6/13 (46%) of non-CMR patients had progressed (*p* = 0.007). Progression-free survival (PFS), as estimated from the date of last drug administration, was significantly longer among CMR patients than non-CMR (log-rank: *p* = 0.001; hazard ratio: 0.127; 95% CI: 0.032–0.511). Two-year PFS was 94% among CMR patients and 62% among non-CMR patients. Univariable Cox regression showed that metabolic response was the only parameter which predicted PFS (*p* = 0.004). Multivariate analysis revealed that metabolic response predicted disease progression (*p* = 0.008). In conclusion, our findings suggest that patients with CMR in an FDG-PET-CT scan may have a favorable outcome even if checkpoint inhibition is discontinued.

## 1. Introduction

Immune checkpoint inhibitors (ICI) are one of the most exciting anti-cancer treatments developed in oncology in the past decade. Since their introduction, they have been used to treat various cancers with great success. Antibodies against cytotoxic T-lymphocyte-associated protein 4 (CTLA-4), programmed cell death protein 1 (PD-1) and programmed cell death 1 ligand 1 (PD-L1) are important state-of-the-art treatments, particularly for patients with metastasized skin cancer. These agents enable T-cells to outrun the escape mechanism of cancer cells and reattack the tumor. Clinical trial results for patients with metastasized melanoma have been impressive. In the Checkmate 067 trial, 52% of patients were alive at 5 years under combined ipilimumab and nivolumab treatment, 44% under nivolumab and 26% under ipilimumab monotherapy [1]. Furthermore, five-year progression-free survival (PFS) was 36% for the combination of ipilimumab and nivolumab, 29% for nivolumab, and 8% for ipilimumab monotherapy [1], leading to a long-term tumor control and potentially curable disease. In fact, 22% of patients treated with ipilimumab and nivolumab achieved a complete response (CR) in a computerized tomography (CT) scan-based follow-up [1]. Meanwhile ICI have proven their benefit in the treatment of many other cancer entities such as renal cell carcinoma [2] and non-small-cell lung cancer [3].

ICI can cause acute and late onset adverse events in varying frequency. Whereas PD-1 antibody monotherapy leads to severe immune-related adverse events (irAE, classified as Common Terminology Criteria for Adverse Events (CTCAE) grade 3 or higher [4]) in about 10% of patients, combined immunotherapy with ipilimumab and nivolumab may affect more than half of the patients [5]. Most of the irAE occur within the first 12 weeks of treatment and are managed with immunosuppressive therapy. However, a considerable amount of irAE appear late in therapy as shown in an analysis from the French MelBase data bank: 43% of patients that were treated with anti-PD1 monotherapy for at least two years developed late onset irAE, 56% in multiple organs [6]. The risk of late onset irAEs increased with the duration of anti-PD1 therapy. Thus, immune checkpoint therapy should only be given as long as necessary and stopped as soon as therapeutically reasonable. However, the optimum duration of treatment, and therefore the best time to stop checkpoint inhibition, is not yet known. It was shown that 86% of patients treated with pembrolizumab for two years and who did not progress had ongoing PFS after treatment discontinuation [7]. Furthermore, in a subgroup analysis of 67 patients within the KEYNOTE-001 study, 24-month disease-free survival was approximately 90% after CR was achieved after at least 6 months of treatment with pembrolizumab and subsequent treatment discontinuation [8].

Treatment of patients with metastasized melanoma is usually monitored by means of regular cross-sectional imaging in the form of a CT scan, magnetic resonance imaging (MRI), or (^18^F)2-fluoro-2-deoxy-D-glucose positron emission tomography (FDG-PET) combined with a low-dose CT scan (FDG-PET-CT scan). No international standard of care exists regarding follow-up imaging. Clinicians decide between the abovementioned techniques based on their specific requirements, local circumstances, and—because of differing costs of the techniques—financial considerations. Unlike CT or MRI, FDG-PET-CT provides functional information on tumor activity [9]. FDG-PET-CT might, therefore, be useful when deciding whether to discontinue checkpoint inhibition [10].

In this study, we retrospectively analyze the outcomes of stage-IV melanoma patients who had an FDG-PET-CT scan when immunotherapy was discontinued for reasons other than tumor progression. Our presented results might be used to help identify patients who might not need further checkpoint inhibition.

## 2. Material and Methods

Patients with metastasized melanoma who have been treated with ICI in clinical routine at 3 German skin cancer centers were included into this retrospective study if the patients (i) had discontinued ICI treatment due to irAEs or their own wishes but not because of progressive disease and (ii) had received an FDG-PET-CT scan just before or within 3 months of treatment discontinuation. In 28 (62%) patients, FDG-PET-CT scan was used for regular disease imaging, in the other 17 patients, an FDG-PET-CT scan was performed in addition to conventional imaging (no more than 12 weeks between imaging techniques) before discontinuation of treatment to get information on disease activity. Disease features, demographics, patient outcomes, and treatment details were collected for each patient from existing medical data. Data collection started in October 2014 and ended in October 2020. The retrospective analysis of the clinical data was approved by the ethical board of the University Hospital Heidelberg (S-454/2015).

**Figure 1:**
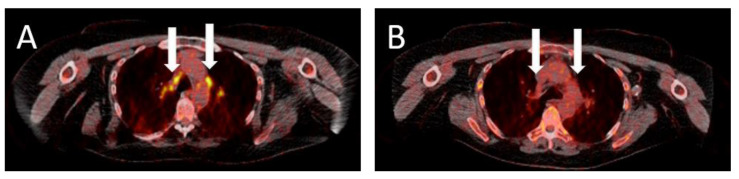
FDG-PET-CT scans of a patient with stage-IV cutaneous melanoma (**A**,**B**). The lymph node metastases showed a complete metabolic response following treatment with combined immunotherapy of ipilimumab 3 mg/kg and nivolumab 1 mg/kg every 3 weeks. (**A**) shows the metastases at the start of immunotherapy. (**B**) shows the same location after 2 administration cycles. Arrows indicate the location of the (former, **B**) metastases.

Consistent with standard clinical practice, CT and MRI scans were analyzed by a radiologist, and FDG-PET-CT scans by a radiologist and a nuclear medicine physician. FDG-PET-CT scans were evaluated using the standard EORTC (European Organisation for Research and Treatment of Cancer [11]) and the inhouse developed PERCIMT (PET response evaluation criteria for immunotherapy [10,12]) criteria and classified as complete metabolic response (CMR), partial metabolic response (PMR), or stable metabolic disease (SMD) in patients with baseline FDG-PET-CT imaging. For patients without a baseline FDG-PET-CT imaging metabolic activity was rated as CMR (no metastases detectable; Figure 1) or non-CMR (metastases detectable). In addition, at the time of FDG-PET-CT scan a liquid biopsy of circulating free tumor DNA was analyzed for melanoma patients with a *BRAF* or *NRAS* mutation, if their plasma was available at the liquid biobank. The use of this material was approved by the ethical board of the University Hospital Heidelberg (S-207/2005). Liquid biopsies were analyzed as described previously, using the Oncomine Colon cfDNA panel (*BRAF* mutation) [13] and the AmpliSeq cancer hotspot panel v2 (*NRAS* mutation) [14] (both Thermo Fisher, Waltham, MA, USA). Targeted next generation sequencing was conducted on an S5 XL sequencing machine (Thermo Fisher, Waltham, MA, USA).

Associations between patient characteristics and metabolic response (CMR or non-CMR) in the FDG-PET-CT scans were determined using univariable analysis. Linear variables were analyzed using the Mann–Whitney U test, whereas categorical variables were analyzed using the chi-square test or likelihood ratio. PFS was defined as the time from the last drug administration to the date of disease progression or death. Patients without progression were censored at the date of last contact. A Kaplan–Meier analysis and log-rank test were performed to assess PFS. PFS was compared between the 2 metabolic response groups by means of a hazard ratio (HR) and 95% confidence interval (CI), based on the Cox proportional hazards regression model. For multivariate analysis, binary logistic regression was used to evaluate the baseline predictors for disease progression and metabolic response status. In addition, univariable Cox regression analysis was performed to determine the baseline predictors of progression-free survival. For all analyses, a two-sided *p*-value of <0.05 was considered statistically significant. IBM SPSS Statistics (SPSS version 25) software (SPSS, Inc., Chicago, IL, USA) was used for statistical analysis.

## 3. Results

### 3.1. Patient Baseline Characteristics

Forty-five patients were included in the study with a median age of 64 (range 34–96) years. Nineteen (42%) patients were female and 26 (58%) were male. Forty-three (96%) patients had metastasized cutaneous and 2 (4%) had metastasized mucosal melanoma. Eighteen (40%) patients had a *BRAF*-mutated and 8 (18%) had an *NRAS*-mutated melanoma. At the start of treatment, the serum tumor marker S100 was elevated in 15 (33%) patients, and 8 (18%) patients had elevated lactate dehydrogenase (LDH) (Table 1). Thirty-five (76%) patients were treated with checkpoint inhibition monotherapy (22 [49%] with pembrolizumab, 9 [20%] with nivolumab, 4 [9%] with ipilimumab) and 10 (22%) patients with combined immunotherapy (nivolumab and ipilimumab).

Twenty (44%) patients were pretreated with either immunotherapy with ICI (38%) or combinations within clinical trials (7%), targeted therapy with either *BRAF*, MEK, or in combination, inhibitors (11%, 4 of 18 patients (22%) with a documented V600 mutation and 1 of 8 patients (12%) with a *NRAS* mutation), or chemotherapy (9%) as first-line treatment.

### 3.2. Tumor Response at Time of Treatment Discontinuation

The median duration of treatment was 21 (range 1–42) months. Sixteen (36%) patients stopped treatment because of irAEs that were grade 3 or higher. The most frequent irAEs were colitis (6 patients), pneumonitis (4 patients), hypophysitis (3 patients), hepatitis (3 patients) and arthritis (2 patients). In FDG-PET-CT scans colitis (4 of 6 patients), pneumonitis (3 of 4 patients), and arthritis (2 of 2 patients) were often detected while hepatitis (0 of 3 patients) and hypophysitis (0 of 3 patients) were diagnosed by clinical assessment and respective blood value deteriorations. Two (4%) patients discontinued treatment because of a secondary malignancy (colon cancer and rectal cancer, respectively). The patient with the colon cancer underwent surgical R0 resection whereas the patient with the rectal cancer received chemotherapy. In both patients neither the melanoma nor the secondary malignancy recurred during follow-up. Another 27 patients (60%) wished to discontinue treatment after discussing the result of the FDG-PET-CT scan with their physician and informed consent (including 2 patients who finished their ipilimumab schedule after 4 cycles). At this time, 32 (71%) patients revealed a complete metabolic response (CMR), the other patients still had metabolic activity in their metastases classifying as non-CMR (Table 2) (9 (20%) patients with partial metabolic response (PMR), 2 (4%) patients with stable metabolic disease (SMD), and 2 (4%) patients with PMR or SMD-not further evaluable because of lacking baseline FDG-PET-CT scan). In the 17 patients who had CT/MRI imaging as routine tumor assessment before FDG-PET-CT scan, 6 patients showed a partial response (PR) and 1 patient had a stable disease (SD) in conventional imaging. Of these, 4 patients with PR and 1 patient with SD were then finally classified as CMR in the following FDG-PET-CT scan.

For the assessment of patient outcomes, patients were grouped based on their FDG-PET-CT scan results into CMR patients (32 patients, 71%) and non-CMR patients (PMR and SMD, 13 patients, 29%) (Table 2).

### 3.3. Patient Survival

**Figure 2:**
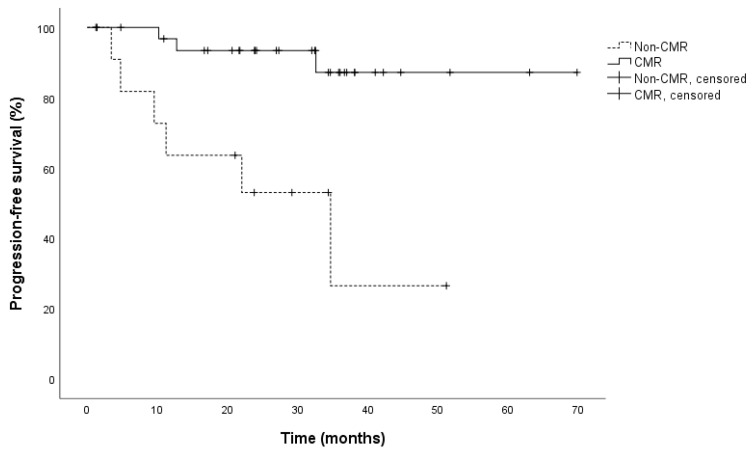
Kaplan–Meier analysis for progression free survival (PFS) in CMR (*n* = 32) and non-CMR patients (*n* = 13) (hazard ratio: 0.127; 95% CI: 0.032–0.511). Median PFS in non-CMR patients was 34.7 months (95% CI: 9.6–59.8), median PFS for CMR patients was not reached (log-rank: *p* = 0.001). CMR, complete metabolic response. CI, confidence interval. PFS, progression-free survival.

The data were analyzed after a median follow-up of almost 3 years (34 months, range 1–70) after treatment discontinuation. At that time, 9 (20%) patients had progressed consisting of 3 of 32 (9%) CMR patients and 6 of 13 (46%) non-CMR patients. This resulted in a significant association between the event of disease progression and the metabolic response status (*p* = 0.007). There was no significant difference in overall survival between CMR and non-CMR patients (*p* = 0.854), as only three patients had died (2 CMR and 1 non-CMR patient). Reasons for death were most likely not due to melanoma: stroke (non-CMR patient), subarachnoid hemorrhage (CMR patient), and senility (CMR patient). Cox regression and Kaplan–Meier analyses revealed that the PFS of CMR patients was significantly longer than that of non-CMR patients (log-rank: *p* = 0.001, HR: 0.127; 95% CI: 0.032–0.511). Median PFS for CMR patients was not reached, median PFS for non-CMR patients was 34.7 months (95% CI: 9.6–59.8; Figure 2). PFS after 2 years was 94% for CMR patients and 62% for non-CMR patients.

Concerning patient baseline characteristics, a significant association was observed between *BRAF* mutation status and metabolic response in univariate analysis (*p* = 0.004) (Table 1). Of the 18 patients with a *BRAF* mutation, 17 achieved a CMR under ICI therapy, whereas only 1 patient experienced non-CMR. Interestingly, the CMR group consisted of more patients with an elevated S100 at treatment initiation (*p* = 0.019). However, there was no significant difference in the frequency of elevated LDH as the more important marker for tumor load (19% CMR group, 15% non-CMR group, *p* = 0.752). In contrast, at the time of FDG-PET-CT scan patients who achieved a CMR had a significantly lower LDH compared to patients with non-CMR (*p* = 0.024) in accordance with the imaging results. Here, only 2 patients revealed an elevated serum S100 with no significant difference between the 2 groups (*p* = 0.292). No significant associations were found between CMR and any of the following patient characteristics: age, sex, *NRAS* mutation status, type of immunotherapy, prior systemic therapy, duration of treatment, and time of follow-up (Table 1). Multivariate analysis revealed that S100 at treatment initiation and *BRAF* status could not predict metabolic response status. However, multivariate analysis showed that metabolic response could predict disease progression (*p* = 0.008) whereas *BRAF* status and LDH values at the time of FDG-PET-CT scan did not show significance. Univariable Cox regression analysis revealed that CMR was the only significant variable to predict PFS (*p* = 0.004). No other variables were rated significant (Table 3).

For two patients with a known mutation in *BRAF* K601E and *NRAS* Q61R, respectively, a liquid biopsy was available at the time of their FDG-PET-CT scan. Both showed a CMR in the FDG-PET-CT scan at treatment discontinuation. Accordingly, no mutated cell-free tumor DNA could be detected in their plasma specimens. Both patients remained relapse-free after a median follow-up of almost four years. 

## 4. Discussion

Metastatic melanoma patients who are treated with ICI and show a CR in CT imaging are known to have a low probability of relapse [7,8,15,16]. In daily practice, however, CR is rarely achieved by means of conventional, morphological imaging, whereas PR is more often diagnosed. Some patients show a stable PR on immunotherapy without substantial CT scan alterations over time. This raises the question of whether other diagnostic imaging techniques such as FDG-PET-CT scans could be used to further evaluate treatment activity and distinguish a residual metastasis from scar tissue. In our analysis, 17 patients underwent FDG-PET-CT after routine CT/MRI imaging. In five patients diagnosed with PR/SD in CT imaging, FDG-PET-CT showed CMR indicating that the melanoma is without activity. Tan et al. [17] compared CT imaging with PET imaging in patients alive one year after the initiation of ICI treatment with PD-1 antibody ± ipilimumab. Here, patients with a CMR in their PET scans were rated as CR in only 28%, PR in 45%, and SD in 2% in CT imaging. Division of patients with a PR in CT imaging based on CMR and non-CMR in PET imaging clearly separated a favorable and a non-favorable patient group concerning risk of recurrence/PFS in Kaplan–Meier analysis. Therefore, metabolic data predicted clinical outcome much better compared to CT imaging. In our study, metabolic response (CMR compared to non-CMR) was the only significant parameter for progression-free survival in univariable Cox regression analysis. Interestingly, other patient baseline characteristics such as tumor load (S100/LDH), age, sex, *BRAF* mutation status, pretreatments, and treatment regime (PD-1 antibody vs. ipi/nivo) did not significantly impact PFS, however this may merely lie in the relatively low patient number of this retrospective study.

Independent of ICI treatment regime, treatment duration, prior systemic therapy, and patient baseline characteristics, this study shows that patients with CMR in FDG-PET-CT scan have a significantly lower risk for disease progression after treatment discontinuation compared to patients not achieving CMR. Therefore, FDG-PET-CT scans might be helpful in deciding whether immunotherapy could be safely discontinued in patients with metastasized melanoma. Most data so far on patients’ clinical outcomes after ICI treatment discontinuation come from clinical trials using CT imaging. Data from the KEYNOTE-006 study showed that approximately 78% of patients who had completed 2 years of pembrolizumab treatment with at least stable disease (SD) in CT/MRI imaging remained progression-free 24 months after treatment cessation. Furthermore, an ongoing response was more often seen in patients achieving CR or PR [18]. A recently published retrospective analysis of 185 patients who had advanced melanoma and discontinued PD-1-antibody treatment without progression or treatment-limiting toxicity, showed that patients in CR at the time of treatment discontinuation were less likely to progress (14%) than patients in PR (32%) or with SD (50%) [16]. In a subgroup analysis of 67 patients within the KEYNOTE-001 trial, patients who achieved CR after 6 months of pembrolizumab treatment and had at least 2 more treatment cycles after diagnosis of CR could discontinue therapy. Notably, the 24-month disease-free survival for these patients after achieving CR was approximately 90% [8]. Another recently published study showed that, 3 years after achieving CR and subsequently discontinuing anti-PD-1 therapy, 72% of metastasized melanoma patients did not relapse [19]. The current data therefore suggest that patients who achieve CR by PD-1 antibody treatment have a good chance of remaining relapse-free [20]. A recently published study assessing tumor response with FDG-PET-CT looked at the responses of melanoma patients after one year of checkpoint inhibition. The authors found that CMR was detected significantly more often in FDG-PET-CT than CR in CT in the same patient at the same time point [17]. Furthermore, patients achieving a CMR stayed progression free in 96% 2 years after CMR detection compared to only 49% with non-CMR. The authors stated that PET scans might be useful for predicting long-term benefits and for guiding the discontinuation of anti-PD-1-based immunotherapy among metastatic melanoma patients after one year of treatment [17]. This is consistent with the results of our study, with 94% of CMR patients staying progression-free compared to 62% of non-CMR patients with CMR in FDG-PET-CT imaging being the only significant predicting factor for PFS; on the other hand, no association was demonstrated between the duration of treatment and CMR. These results might help to pave the way towards a more standardized follow-up procedure, including the performance of FDG-PET-CT scans, particularly when immunotherapy discontinuation is being considered in patients not achieving a CR in CT imaging. The use of FDG-PET-CT for regular tumor assessment in patients with metastasized melanoma under immunotherapy remains the subject of current investigation [10].

Cell-free circulating tumor DNA can be used as a source of liquid biopsy for cancer patients. It has high potential as a method for assessing tumor progression and identifying targets for therapy, potentially being an additional instrument for evaluating clinical response to treatment [21]. Because our study was retrospective, we were able to assess the plasma specimens of only two patients at the time of FDG-PET-CT scanning. No tumor DNA was found in either blood sample, which is consistent with CMR in the FDG-PET-CT scan before treatment discontinuation. Furthermore, neither patient relapsed later. However, since specimens were taken at only one time point, no conclusions can be drawn concerning dynamics or trends over time. Moreover, further studies are required to evaluate the suitability of liquid biopsies as a means of evaluating tumor responsiveness in stage-IV melanoma patients treated with immunotherapy [22]. Liquid biopsies might be an additional tool to safely diagnose a complete tumor remission before stopping immunotherapy.

### Limitations

Limitations of the study especially include its retrospective nature and the limited number of patients (45). In addition, only 62% of patients (28) had a baseline PET-CT scan available for direct comparison. Included patients were heterogeneous (as they are in routine treatment) from their baseline characteristics, the different ICI regimes used, different pretreatments and different reasons for treatment discontinuation leading to variable treatment lengths. However, in our study, the duration of treatment was not a significant factor for achieving a CMR. In univariable Cox regression analysis neither baseline characteristics such as the *BRAF* status or the tumor load at treatment start (LDH/S100) nor the pretreatments and therapy regimes used were significantly influencing PFS with metabolic response as the only significant factor. In a recent pooled analysis of the CheckMate 069/067 and 066 trials in patients who achieved a CR in CT/MRI staging after treatment with nivolumab ± ipilimumab patients discontinued therapy for different reasons, also including toxicity, patient request, and maximum benefit. The analysis showed that patients with a CR had the same progression free and overall survival independent of the treatment that led to the CR [23].

## 5. Conclusions

Our results suggest that patients achieving a CMR under immune checkpoint inhibition may have a good clinical outcome, even without further immunotherapy. However, the need for further studies in the form of prospective, multicenter trials to confirm our preliminary results is mandatory.

## Figures and Tables

**Table 1:** Patient characteristics.

Patient Characteristics	CMR Group	Non-CMR Group	All Patients	*p*-Value
	(*n* = 32)	(*n* = 13)	(*n* = 45)	
Age (years)				0.94
Median	66	62	64	
Range	34–84	47–96	34–96	
Sex				0.734
Female	13 (40%)	6 (46%)	19 (42%)	
Male	19 (60%)	7 (54%)	26 (58%)	
*BRAF* mutation	17 (53%)	1 (8%)	18 (40%)	**0.004**
*BRAF* V600E	13 (41%)	1 (8%)	14 (31%)	
*BRAF* V600K	3 (9%)	–	3 (7%)	
*BRAF* K601E	1 (3%)	–	1 (2%)	
Wild type	15 (47%)	11 (84%)	26 (58%)	
Missing	–	1 (8%)	1 (2%)	
*NRAS* mutation				0.484
p.Q61(R/K/L)	5 (15%)	3 (24%)	8 (18%)	
Wild type	27 (85%)	9 (68%)	36 (80%)	
Missing	–	1 (8%)	1 (2%)	
S100 at start of treatment				**0.019**
Elevated	14 (44%)	1 (8%)	15 (33%)	
Normal	14 (44%)	10 (77%)	24 (53%)	
Missing	4 (12%)	2 (15%)	6 (14%)	
S100 at time of FDG-PET-CT scan				0.292
Elevated	2 (6%)	–	2 (4%)	
Normal	24 (75%)	8 (62%)	32 (71%)	
Missing	6 (19%)	5 (38%)	11 (25%)	
			
LDH at start of treatment				0.752
Elevated	6 (19%)	2 (15%)	8 (18%)	
Normal	26 (81%)	11 (85%)	37 (82%)	
Missing	–	–	–	
LDH at time of FDG-PET-CT scan				**0.024**
Elevated	–	2 (16%)	2 (4%)	
Normal	31 (97%)	10 (76%)	41 (92%)	
Missing	1 (3%)	1 (8%)	2 (4%)	
Therapy				0.502
PD-1 antibody	23 (72%)	8 (61%)	31 (69%)	
Ipilimumab	3 (9%)	1 (8%)	4 (9%)	
Ipilimumab + nivolumab	6 (19%)	4 (31%)	10 (22%)	
Prior systemic therapy	15 (47%)	5 (38%)	20 (44%)	0.607
ICI	12 (38%)	5 (38%)	17 (38%)	
Targeted therapy	5 (16%)	–	5 (11%)	
Chemotherapy	3 (9%)	1 (8%)	4 (9%)	
Study treatment	3 (9%)	–	3 (7%)	
Duration of treatment (months)				0.468
Median	22	16	21	
Range	1–42	1–34	1–42	
Follow-up (months)				0.764
Median	34	33	34	
Range	5–70	1–57	1–70	
Reason for discontinuation				
Wish of patient	22 (69%)	5 (38%)	27 (60%)	
irAEs	9 (28%)	7 (54%)	16 (36%)	
Secondary malignancies	1 (3%)	1 (8%)	2 (4%)	
Disease progression	3/32 (9%)	6/13 (46%)	9/45 (20%)	**0.007**

Patient characteristics. The *p*-values in Table 1 are from univariable analysis. Linear variables were analyzed using the Mann–Whitney U test and categorial variables were analyzed using the chi-square test or likelihood ratio. *p* < 0.05 = statistically significant (marked in bold). CMR, complete metabolic response; FDG-PET-CT scan, (^18^F)2-fluoro-2-deoxy-D-glucose positron emission tomography combined with a low-dose CT scan LDH, lactate dehydrogenase; ICI, immune checkpoint inhibition (with pembrolizumab, nivolumab, ipilimumab or ipilimumab + nivolumab); targeted therapy (with MEKi, BRAFi ± MEKi ± ICI); chemotherapy (with bleomycin, temozolomide or dacarbazine); study treatment (with taldalafil or durvalumab + tremelimumab + tebentafusp); irAEs, immune-related adverse events.

**Table 2:** Evaluation of FDG-PET-CT scan and of preceding CT/MRI at time of therapy discontinuation.

Characteristic	All Patients	Patients with Preceding CT/MRI Imaging	
	*n* = 45	*n* = 17	
FDG-PET-CT Response		FDG-PET-CT Response	CT/MRI Response
CMR	32/45 (71%)	CMR 15/17 (88%)	CR 10/17 (59%)
Non-CMR	13/45 (29%) *	Non-CMR 2/17 (12%) *	PR 6/17 (35%)
			SD 1/17 (6%)

CMR, complete metabolic response; PMR, partial metabolic response; SMD, stable metabolic disease; CR, complete response; PR, partial response; SD, stable disease. * As the 17 patients with routine CT/MRI imaging do not have a baseline FDG-PET-CT, response can only be evaluated as CMR and non-CMR in these patients.

**Table 3:** Univariable Cox regression analysis for progression-free survival.

Patient Characteristic	Univariable Cox Regression Analysis	
	HR (95% CI)	*p*-Value
Age (years)	1.2 (0.3–4.6)	0.746
Sex (male compared with female)	0.8 (0.2–3.1)	0.720
*BRAF* (mutation compared with wild type)	2.6 (0.5–12.7)	0.230
*NRAS* (mutation compared with wild type)	31.4 (0.1–26,703.8)	0.316
S100 baseline (normal compared with elevated)	1.1 (0.3–4.4)	0.889
S100 time of PET-CT (normal compared with elevated)	21.2 (0.0–27,689,946,664.3)	0.775
LDH baseline (normal compared with elevated)	0.7 (0.2–3.6)	0.706
LDH time of PET-CT (normal compared with elevated)	20.9 (0.0–12,520,603,321.5)	0.768
Therapy (anti-PD-1 compared with ipi ± nivo)	0.9 (0.2–3.9)	0.974
Prior systemic therapy (yes compared with no)	1.4 (0.3–5.6)	0.651
Metabolic response (CMR compared to non-CMR)	7.9 (1.9–31.7)	**0.004**

HR, hazard ratio; CI, confidence interval; anti-PD-1, programmed cell death protein 1 antibody; ipi, ipilimumab; nivo, nivolumab; *p* < 0.05 = statistically significant (marked in bold).

## Data Availability

The data presented in this study are available on request from the corresponding author. The data are not publicly available due to privacy and ethical reasons.
